# REV7: a small but mighty regulator of genome maintenance and cancer development

**DOI:** 10.3389/fonc.2024.1516165

**Published:** 2025-01-07

**Authors:** Lara R. Maggs, Mitch McVey

**Affiliations:** Department of Biology, Tufts University, Medford, MA, United States

**Keywords:** REV7, genome stability, DNA damage response, cancer, chemotherapeutic resistance, chromatin

## Abstract

REV7, also known as MAD2B, MAD2L2, and FANCV, is a HORMA-domain family protein crucial to multiple genome stability pathways. REV7’s canonical role is as a member of polymerase ζ, a specialized translesion synthesis polymerase essential for DNA damage tolerance. REV7 also ensures accurate cell cycle progression and prevents premature mitotic progression by sequestering an anaphase-promoting complex/cyclosome activator. Additionally, REV7 supports genome integrity by directing double-strand break repair pathway choice as part of the recently characterized mammalian shieldin complex. Given that genome instability is a hallmark of cancer, it is unsurprising that REV7, with its numerous genome maintenance roles, is implicated in multiple malignancies, including ovarian cancer, glioma, breast cancer, malignant melanoma, and small-cell lung cancer. Moreover, high REV7 expression is associated with poor prognoses and treatment resistance in these and other cancers. Promisingly, early studies indicate that REV7 suppression enhances sensitivity to chemotherapeutics, including cisplatin. This review aims to provide a comprehensive overview of REV7’s myriad roles in genome maintenance and other functions as well as offer an updated summary of its connections to cancer and treatment resistance.

## Introduction

**Figure 1:**
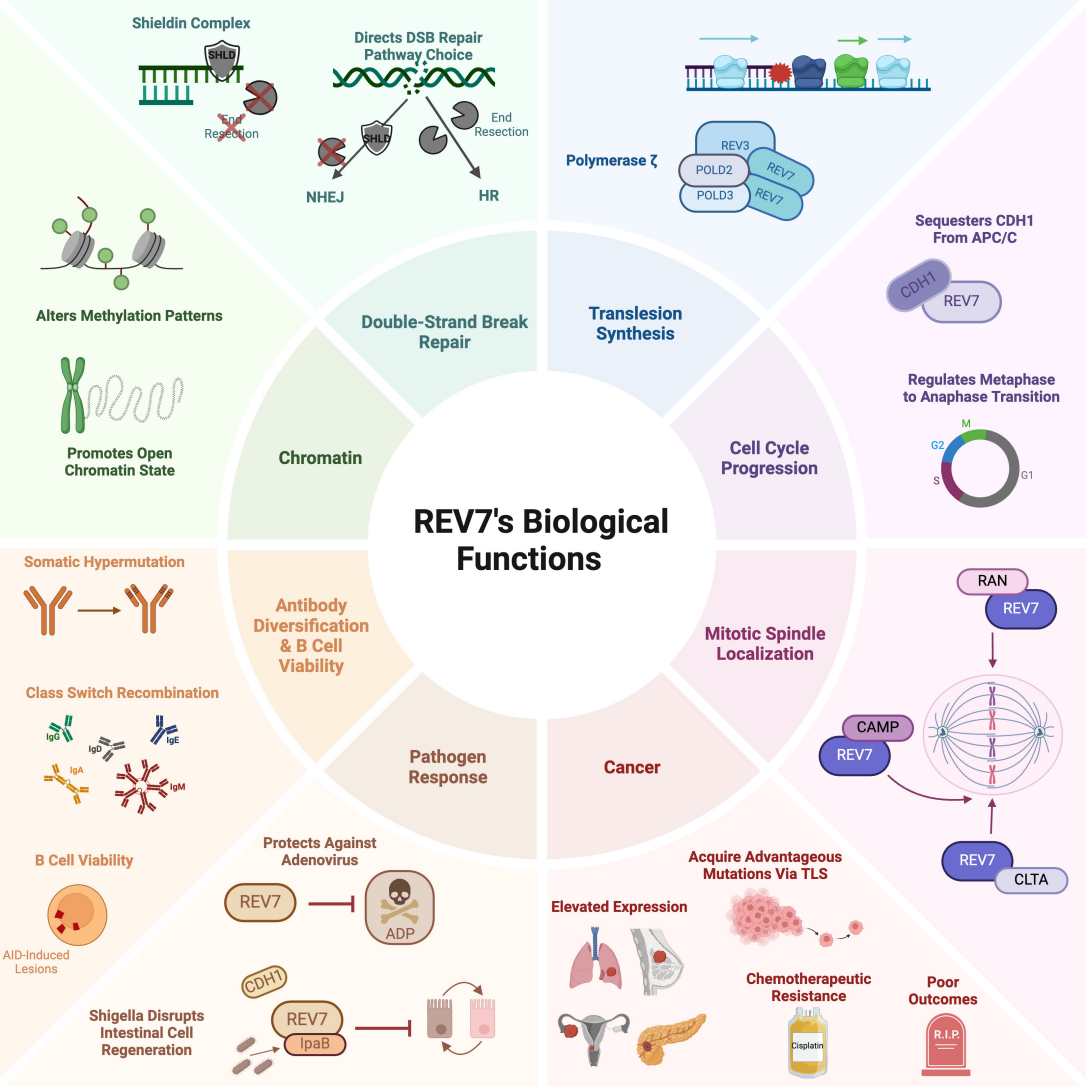
REV7’s biological functions.

Despite its small size of just 211 amino acids, REV7 plays an outsized role in promoting genome stability and cell viability. It participates in several DNA damage response pathways, including translesion synthesis, interstrand crosslink repair, and double-strand break repair. As a component of the shieldin complex, REV7 regulates double-strand break repair pathway choice by protecting single-stranded DNA from nucleolytic processing, thereby promoting repair via non-homologous end joining. Additionally, REV7 functions in cell cycle regulation, antibody diversification, B cell survival, and primordial germ cell development. Given its involvement in myriad cellular processes, it is not surprising that REV7 has a substantial impact on cancer biology. Clinically, REV7 is implicated in various cancers, including lung, ovarian, and skin cancers (**Table 1**). Furthermore, REV7 contributes to chemotherapeutic resistance, making it an appealing target for pharmacologic inhibition to enhance the sensitivity of cancer cells to chemotherapy. In this review, we present a thorough examination of the mechanisms by which REV7 impacts genome stability and other biological functions via its interactions with multiple effector proteins, along with a current summary of its connections to cancer and chemotherapeutic resistance (**Figure 1**).

**Table 1:** REV7 is associated with numerous cancers.

Organ	Cancer Type	Reference
Bladder	Bladder	(173)
Brain	Glioma	(168, 169, 220)
Breast	Breast	(174)
	Triple negative	(157)
Cervix	Cervical	(166)
Colon	Colorectal	(163–165)
Esophagus	Esophageal squamous cell	(175)
Hematopoietic	Diffuse large B-cell lymphoma	(158)
Lung Lung	Small cell	(172, 211)
	Non-small cell	(171)
Ovary	Ovarian	(182)
	Clear cell carcinoma	(181)
Pancreas	Pancreatic ductal adenocarcinoma	(160)
Pharynx	Nasopharyngeal carcinoma	(167)
Skin	Basal cell carcinoma	(159)
	Melanoma	(159)
	Squamous cell carcinoma	(159)
Testicle	Testicular germ cell	(161, 162)

## Conformational changes of the REV7 HORMA domain protein drive its function in multiple cellular pathways

REV7 was first identified in a genetic screen of *Saccharomyces cerevisiae* mutants sensitive to ultraviolet radiation (1). Mutants lacking REV7 showed deficient reversion of UV damage at the *LYS2* locus; hence, the gene was named *Rev7* and joined the “reversionless” family alongside *Rev1, Rev2, Rev3*, *Rev4, Rev5, and Rev6* (1, 2). As REV7 accumulated distinct biological roles, it earned several additional names. Sequence homology (26% identity) with the spindle assembly checkpoint protein MAD2 added MAD2B, MAD2L2, and MAD2β to REV7’s list of monikers (3–5). Whole exome sequencing of a Fanconi anemia patient identified inactivating biallelic mutations in REV7 as one cause of the disorder, earning REV7 the name FANCV (6). REV7 will be used henceforth.

### The HORMA domain

REV7 belongs to the HORMA domain protein family. Human REV7 has 211 amino acids, 191 of which constitute the HORMA domain (7). The HORMA domain is an evolutionarily conserved, versatile protein-protein interaction module required for various eukaryotic signaling pathways. It was initially identified during a systematic comparative analysis of yeast DNA repair proteins, where a conserved domain was observed in Hop1, Rev7, and Mad2 (8). Accordingly, the region was designated the HORMA domain. Subsequent work identified HORMA domains in p31^comet^, a spindle assembly checkpoint inhibitor, and in the autophagy proteins ATG13 and ATG101 (9–11). The HORMA domain’s unique structural features make these proteins attractive interaction partners. As such, this domain is a common element in proteins involved in mitotic checkpoints, chromosome synapsis, autophagy, and the DNA damage response, with more interactors likely remaining unidentified (12).

**Figure 2:**
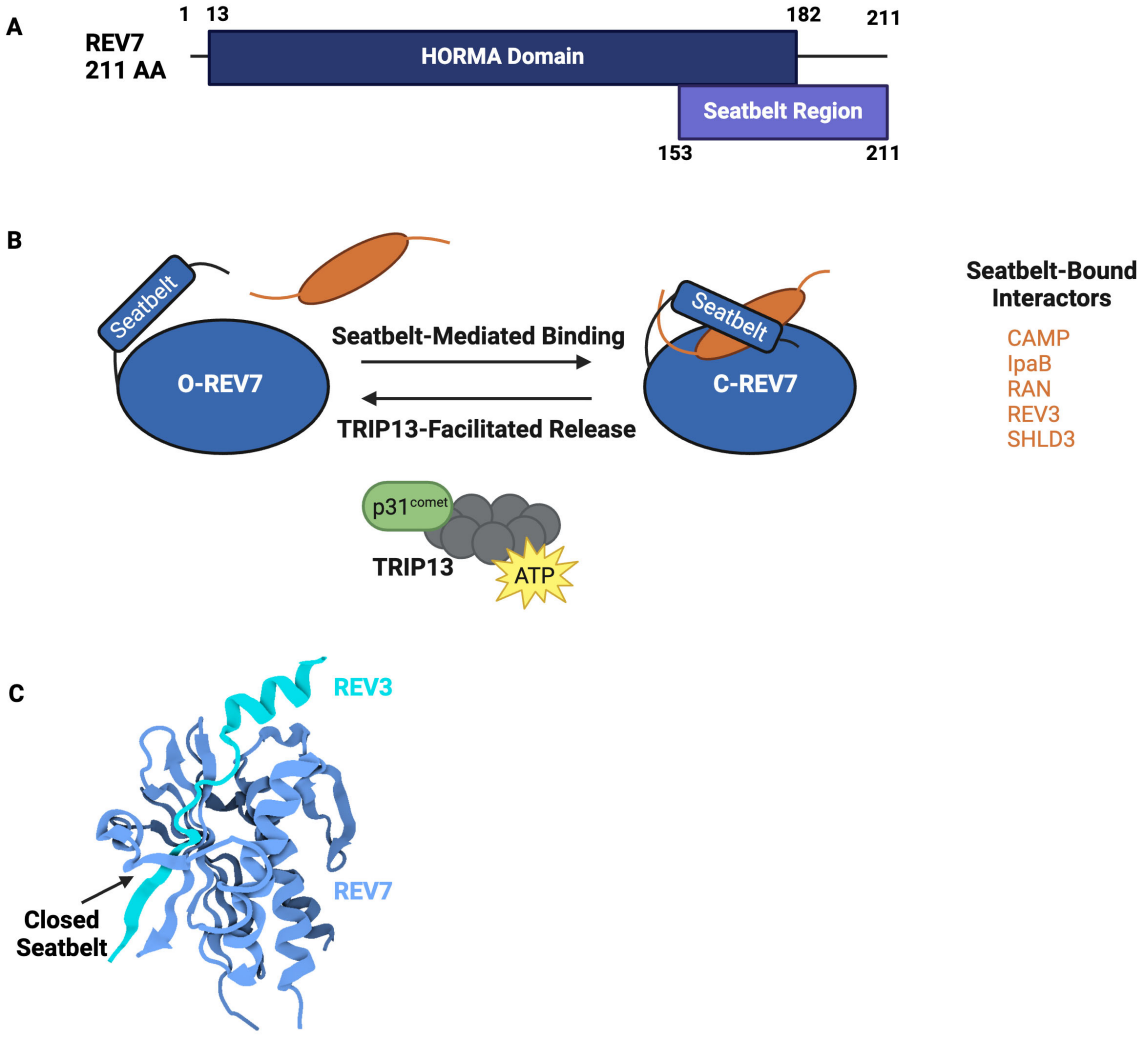
REV7’s HORMA Domain Facilitates Interactions with Various Complexes. **(A)** Domain structure of REV7. REV7 is 211 amino acids and is primarily composed of a HORMA domain. The C-terminus seatbelt region is structurally dynamic and used to bind interactors. **(B)** Schematic representation of conformational remodeling driving REV7 activity. In the inactive state, REV7’s seatbelt is open and unbound (O-REV7) whereas in the active state, REV7’s seatbelt binds a peptide, closing (C-REV7) around it to create a highly stable complex. TRIP13 catalyzes interactor release, thereby returning C-REV7 to O-REV7. Additional interactors bound by REV7’s seatbelt include CHAMP1, IpaB, RAN, REV3, and SHLD3. **(C)** Crystal structure of REV7 in complex with REV3 (PDB 6BI7) (14). REV7 (blue) entraps a REV3 fragment (cyan) via seatbelt-mediated binding in Pol ζ. REV7’s closed seatbelt region is denoted by an arrow.

The HORMA domain is approximately 200 amino acids and is composed of a core region and a C-terminal seatbelt region (**Figure 2A**) (13). The structurally unique, flexible seatbelt region associates with the HORMA core domain in two distinct conformations, creating an open or closed state. In the open state, the seatbelt is unengaged and flush to the core region. In the closed state, the seat belt extends across the core region and wraps itself around an interacting peptide. These conformational transformations create a topological linkage between the HORMA domain and binding partners, locking the interacting peptide within the seat belt region and creating an exceptionally stable complex (**Figures 2B, C**). Human REV7 utilizes seatbelt-mediated entrapment when interacting with many of its binding partners, including REV3, CAMP, IpaB, RAN, and SHLD3 (**Table 2**) (14–17). While not validated via crystal structure, ELK-1, TCF4, and MDC are predicted to also interact with REV7 via the seatbelt region (18, 19). An alternate binding interface is used during REV7 homodimerization as well as during REV7’s interactions with MAD2, REV1, SHLD2, CDH1, TRIP13, and p31^comet^ (14). REV7’s seatbelt engages the interacting protein’s REV7-binding motif (RBM). The consensus sequence for the RBM is ϕϕxPxxxpP, in which ϕ is an aliphatic residue, x is any residue, and P/p is proline, with P>p for binding affinity (20).

**Table 2:** Known REV7 interactors.

Protein	Function	Organism	Detection Method	Reference
53BP1	DDR, NHEJ	H	MS, CoIP	(221)
911 Complex	DDR	Y	Y2H, P, CoIP	(222)
ADP	Adenovirus infection, cell lysis	H	Y2H, P, CoIP	(132)
CDC20	APC/C, cell cycle regulation	H	Y2H, P, Co-IP	(82, 138, 223)
CDC27	APC/C, cell cycle regulation	H	P, CoIP	(223)
CDH1	APC/C, cell cycle regulation	H	P, CoIP	(5, 83, 84)
CDK1	Cell cycle regulation, PGC	M	CoIP	(105)
CAMP/CHAMP1	Cell cycle regulation, chromosome segregation	H	CoIP, MS, XRD	(15, 90, 224)
CLTA	Cell cycle regulation, chromosome segregation	H	Y2H, P, CoIP	(91, 225)
CST-Complex	DDR, NHEJ	H	Y2H, CoIP	(69)
DDK	TLS	Y	CoIP	(226)
ELK-1	Transcription factor	H	Y2H, P, CoIP	(19)
EspF	*E. coli* virulence effector	EC	Y2H	(139)
G9A	Epigenetics, histone methyltransferase	M	CoIP	(105)
GLP	Epigenetics, histone methyltransferase	M	CoIP	(105)
HCCA2	Transcriptional coactivator	H	Y2H, P, CoIP	(227)
HR23B	DDR, NER	H	MS, CoIP	(228)
IpaB	Cell cycle disruption by *Shigella*	H	Y2H, P, CoIP, XRD	(16, 138)
MAD2	SAC, mitosis	H	Y2H, P, XRD	(4, 14)
MDC9/ADAM9	Mitosis	H	Y2H, P	(229)
NCOA3	Transcriptional coactivator	H	CoIP, MS	(165)
p31(comet)	Cell cycle regulation, SAC	H	Y2H, CoIP, XRD	(14, 22)
p53	DDR, DSB	H	P	(230)
POLD2/Pol31	TLS, Pol ζ	Y, H	Cryo-EM	(17)
POLD3/Pol32	TLS, Pol ζ	Y, H	P, Cryo-EM	(17, 231)
PRCC	Cell cycle regulation	H	Y2H, FRET, CoIP	(232)
PRDX2	Antioxidant, carcinogenesis	H	CoIP, MS	(175)
RAN	Cell cycle regulation	H	Y2H, P, CoIP, XRD	(16, 233)
REV1	TLS	Y, M, H	Y2H, P, CoIP, Cryo-EM, XRD	(44, 234–239)
REV3	TLS, Pol ζ	Y, M, H	Y2H, P, CoIP, Cryo-EM, XRD	(14, 235–237, 240, 241)
REV7	TLS, Pol ζ, DSB	Y, M, H	Y2H, CoIP, NMR	(14, 68, 242)
SHLD1	DSB, NHEJ	M, H	Y2H, P, Cryo-EM, MS, XRD	(66, 70, 73, 243–245)
SHLD2	DSB, NHEJ	M, H	Y2H, P, Cryo-EM, MS, XRD	(66, 70, 73, 244–247)
SHLD3	DSB, NHEJ	M, H	Y2H, CoIP, Cryo-EM, XRD	(73, 242, 246, 248, 249)
SIM2	Transcription factor	R, H	Y2H, CoIP	(225)
TCF4	Transcription factor	H	Y2H, P, CoIP	(18, 250)
TFII-I	Transcription factor	H	P, CoIP, MS	(251)
TRIP13	Cell cycle regulation, chromosome segregation, SAC	H	CoIP, MS, Cryo-EM, XRD	(21, 22, 68, 246)

REV7 interacts with proteins that perform a wide variety of functions. DDR, DNA damage response; NHEJ, non-homologous end joining; APC/C, anaphase-promoting complex/cyclosome; TLS, translesion synthesis; SAC, spindle assembly checkpoint; DSB, double-strand break; PGC, primordial germ cell. Interactions detected via co-immunoprecipitation (CoIP), fluorescence resonance energy transfer (FRET), GST-tagged pull-down (P), mass spectrometry (MS), X-ray diffraction (XRD), chromatin immunoprecipitation (ChIP), and/or yeast two-hybrid (Y2H). Organisms studied include human (H), mouse (M), rat (R), yeast (Y), and *E. coli* (EC).

### REV7 activity is controlled by stable conformational changes

Changes in HORMA protein conformation alter protein-protein interactions, indicating that complex assembly and disassembly are dictated by structural rearrangements. Conformational remodeling from the active, closed state to the inactive, open state requires seatbelt disengagement. Seatbelt disengagement releases the interacting peptide and returns the HORMA domain to an inactive state. This process is energy-intensive and is catalyzed by TRIP13, an ATPase, and its adapter, p31^comet^. TRIP13-p31^comet^ interacts directly with REV7 and disengages the seatbelt domain, thus releasing the entrapped binding partner (21, 22). For example, TRIP13-p31^comet^ remodeling inactivates Polymerase ζ and shieldin, inhibiting translesion synthesis and non-homologous end joining, respectively, by causing these complexes to disassemble (21). This disassembly allows subsequent processes to proceed. While the mechanism by which REV7’s seatbelt is released is well-characterized, the process underlying REV7’s transition to a closed state remains poorly understood and requires further investigation.

### Additional REV7 properties encourage protein-protein interaction

REV7’s ability to reshape itself to facilitate interaction with diverse RBMs allows for expansive potential interactions. In addition to the characteristic seatbelt interactions shared by all HORMA proteins, HORMA domain proteins have a propensity to dimerize using a flexible dimerization interface. REV7 can undergo both homodimerization and heterodimerization at this interface (14). REV7 homodimerization is essential to the assembly of the REV1-Pol ζ complex and is necessary for the direct replication of damaged bases via translesion synthesis (14). Anaphase promoting complex/cyclosome (APC/C) inhibition and double-strand break repair also require REV7 homodimerization (23).

Additionally, REV7’s dimerization interface is used when binding fellow HORMA domain proteins, MAD2 and p31^comet^ (14). As p31^comet^ is required for seatbelt disengagement, heterodimerization is implicated in complex disassembly (14). Beyond properties shared with other HORMA domain proteins, REV7 possesses further characteristics that make it an excellent binding partner. Structural analysis of REV7 in complex with RAN, IpaB, REV3, and CAMP revealed a dynamic adapter region within the seatbelt domain (16). This region undergoes secondary structure rearrangement to accommodate assorted RBMs.

## REV7 acts in myriad DNA damage response pathways

DNA serves as the template for the fundamental processes of life: replication and transcription. The preservation of genome stability is imperative for health and viability. Maintaining genome stability requires both high-fidelity replication and a robust DNA damage response (DDR). As such, organisms have developed highly sophisticated mechanisms to repair or tolerate DNA damage. This is no small task, as the genome is constantly under attack by both endogenous (e.g., hydrolysis, base mismatches) and exogenous insults (e.g., ultraviolet radiation, ionizing radiation). Unrepaired damage threatens cell viability and genome stability, making it a significant contributor to cell death, aging, and carcinogenesis. Therefore, it is crucial that cells swiftly and efficiently respond to DNA damage through an elaborate network of DDR pathways. The DDR is bifurcated into repair, in which lesions are removed and replaced with correct information, and tolerance, in which lesions are bypassed through potential error-prone replication. REV7 plays a role in both damage tolerance and damage repair pathways, as described below.

### REV7 facilitates translesion synthesis as a critical component of polymerase ζ

When challenged by DNA lesions, standard replicative polymerases are frequently unable to continue synthesis. This is deleterious to genome stability as unrepaired lesions can stall replication forks, create gaps, cause replication fork collapse, and induce mutations. Common replication-blocking adducts include abasic sites, T-T photoadducts, and T-T cis-syn cyclobutane dimers (24).

Translesion synthesis (TLS) is a conserved damage tolerance mechanism in which specialized polymerases tolerate these lesions by facilitating their direct replication (25–28). TLS polymerases include the Y-family polymerases REV1, Pol κ, Pol η, and Pol ι, the B-family Pol ζ, and the A-family polymerase Pol θ (17, 29). As with many DDR pathways, TLS is a double-edged sword. It protects against cytotoxic events like fork stalling and fork collapse at the expense of an increased mutation rate. Indeed, TLS is the principal contributor to spontaneous mutagenesis in cells (30–32). Translesion synthesis polymerases are less processive and demonstrate lower fidelity than standard replicative polymerases (26). This low fidelity is due to these polymerases’ diminished ability to discriminate between accurately paired nucleotides and their lack of 3′–5′ exonucleolytic proofreading (33, 34).

**Figure 3:**
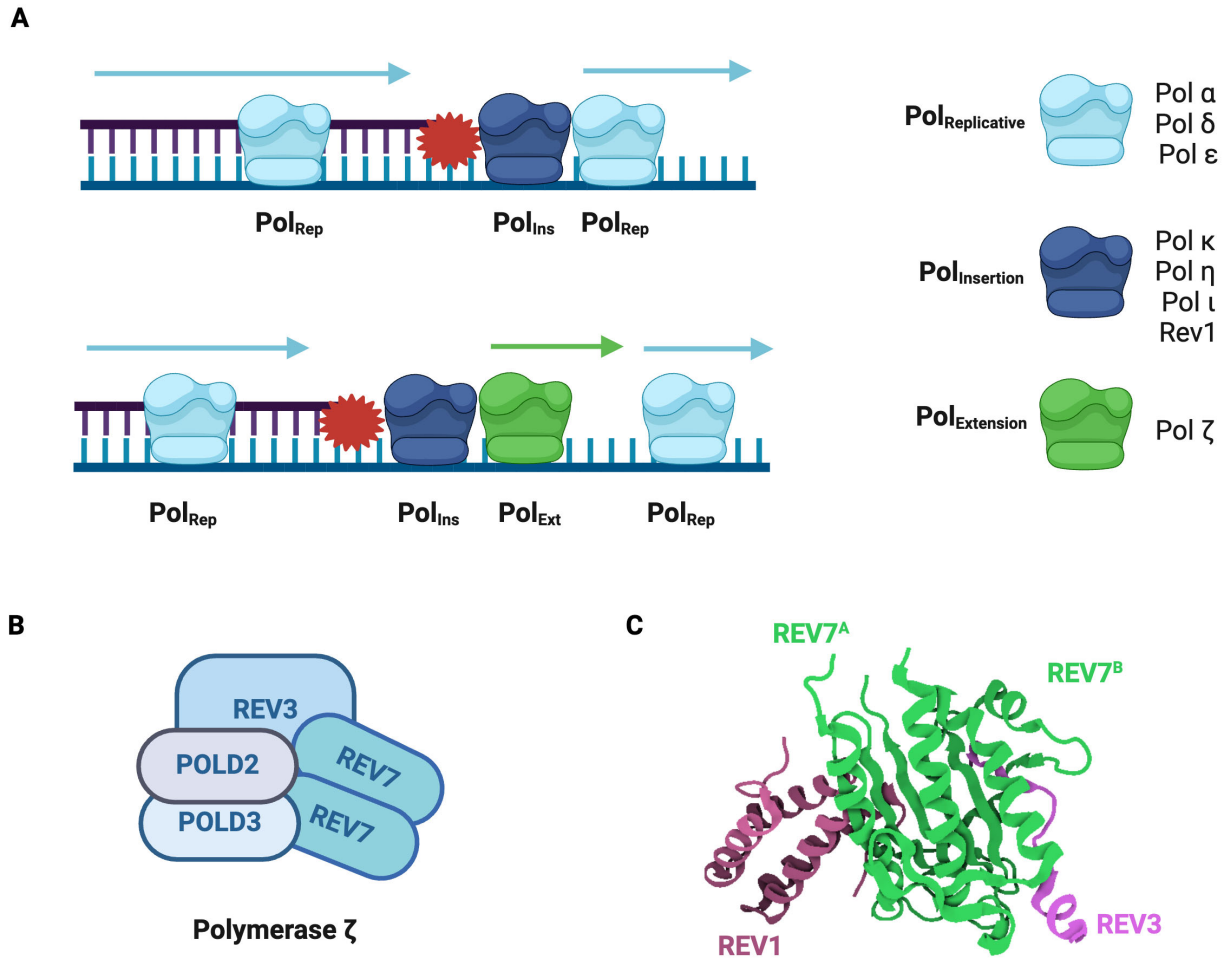
Polymerase ζ is a Specialized Translesion Synthesis Polymerase. **(A)** Model for DNA lesion bypass via one-step or two-step translesion synthesis. In one-step TLS, a standard replicative polymerase is transiently replaced by a specialized insertion polymerase, which introduces a nucleotide opposite the damaged base and is then replaced by the original replicative polymerase. In two-step TLS, an extension polymerase extends from the nucleotide inserted by the insertion polymerase and then the standard replicative polymerase resumes synthesis. **(B)** Pol ζ is composed of REV3, POLD2, POLD3, and a REV7 dimer. REV3 is the catalytic subunit. **(C)** Structure of the Pol ζ-REV1 mutasome. REV7 (green) homodimer in complex with REV3 fragment (pink) and REV1 fragment (purple) (PDB: 3VU7) (44). The REV7 dimer is the core of the Pol ζ-REV1 mutasome and is required for its function.

Due to its mutagenic nature, the switch from a standard replicative polymerase to a TLS polymerase is tightly regulated. One mechanism by which this occurs is through the monoubiquitination of the replication factor proliferating cell nuclear antigen (PCNA) (35). This induces the recruitment of TLS polymerases that interact directly with ubiquitinated PCNA (36). Another recruitment mechanism involves the REV1 protein, which binds to multiple TLS polymerases using its C-terminus (37). Following TLS recruitment, the subsequent direct bypass of a DNA lesion occurs through either a one-step or two-step process (**Figure 3A**). In one-step TLS, a single specialized polymerase inserts a nucleotide opposite the lesion and a standard replicative polymerase extends from the distorted termini. REV1 is frequently employed in one-step TLS, as it has deoxycytidine monophosphate (dCMP) transferase activity and specifically inserts cytosine opposite template lesions (38). In two-step TLS, specialized polymerases are sequentially recruited to the lesion. The first polymerase (Pol_Ins_) inserts a nucleotide opposite the damaged base and the second polymerase (Pol_Ext_) extends the distorted termini (26). The majority of TLS is of the two-step variety in which Polymerase ζ frequently acts as the Pol_Ext_.

Polymerase ζ (Pol ζ) is a B-family DNA polymerase composed of REV3, POLD2, POLD3, and a REV7 dimer (**Figure 3B**) (25, 39, 40). Genetic and biochemical studies demonstrate that Pol ζ can insert nucleotides opposite lesions, including damaged nucleotides, pyrimidine dimers, and abasic sites (38, 41, 42). In the REV1-Pol ζ mutasome, REV1 not only inserts a nucleotide across the lesion but also recruits and acts as a scaffold for Pol ζ through its C-terminal domain (43). When in complex with Pol ζ, REV1 interacts directly with REV7 (**Figure 3C**) (44). Both REV7 subunits use their seatbelt region to entrap REV3, Pol ζ’s catalytic subunit, thus forming a stable complex (17, 44).

While TLS is inherently an error-prone process, Pol ζ exerts particularly mutagenic activity. In yeast, 95% of base pair damage induced by UV radiation is due to Pol ζ (31). Moreover, Pol ζ is responsible for the vast majority of spontaneous mutagenesis in yeast and the deletion of Pol ζ subunits reduces spontaneous mutation rates up to 80% (45). In human cells, REV1-Pol ζ is responsible for more than half of spontaneous mutagenesis (32). Consequently, REV7 is implicated in carcinogenesis and chemotherapeutic resistance, which will be reviewed in depth in forthcoming sections.

### REV7 belongs to the Fanconi anemia V complementation group

Fanconi anemia (FA) is a rare multi-system disorder characterized by various congenital abnormalities, severe bone marrow failure, hypersensitivity to crosslinking agents, and a significantly increased predisposition to solid tumors and hematological malignancies. Compared to the general population, individuals with FA have a 40-fold higher risk of developing any solid tumor and a 700-fold higher risk of developing acute myelogenous leukemia (46). This disorder is caused by biallelic mutations in any of the 22 identified Fanconi anemia complementation group (FANC) genes (47). These genes encompass a range of DDR functions, including replication fork stabilization, homologous recombination, nucleotide excision repair, and TLS (48–50). The FANC genes are united in their essential contributions to replication fork stability and interstrand crosslink (ICL) repair. ICLs occur when a covalent link forms between bases on complementary strands of the double helix. These are particularly hazardous adducts, as they prevent DNA strand separation, thus hindering both transcription and replication. The FA pathway orchestrates ICL repair via an intricate process that enlists multiple distinct DDR pathways including homologous recombination, nucleotide excision repair, and TLS (51, 52).

REV7 is a recent addition to the FANC gene family. Its initial link to FA was established by a young patient who presented with archetypal FA features, including severe bone marrow depletion and a positive chromosome breakage test. Sequencing of known FANC genes returned no findings, indicating her disease was due to a mutation in an uncharacterized FANC gene. Whole exome sequencing identified biallelic inactivating mutations in REV7 (6). Subsequent studies in patient-derived cells and CRISPR/Cas9 REV7 knockout cells recapitulated the FA phenotypes, confirming that REV7 acts in the FA pathway. In light of these findings, REV7 joined the growing ranks of FANC genes as FANCV. It remains unknown which of REV7’s roles are responsible for the FA phenotype.

### Shieldin directs double-strand break repair pathway choice

Double-strand breaks (DSBs) are an exceptionally cytotoxic form of DNA damage and readily threaten cell viability and genome stability. Unrepaired DSBs can trigger apoptosis and provoke gross chromosomal rearrangements, including deletions, translocations, and amplifications (53). This notably pernicious form of DNA damage is predominantly repaired by non-homologous end joining (NHEJ) and homologous recombination (HR) (53, 54). NHEJ, active throughout the cell cycle, is the dominant DSB repair pathway and accounts for approximately 80% of DSB repair in human cells (55, 56).

In NHEJ, broken DSBs ends are recognized and bound by Ku70/80, which scaffolds and recruits a complex of core and accessory NHEJ components (57, 58). This complex brings together the broken DNA ends and directly ligates them, repairing the break (59–61). If ends aren’t immediately compatible for ligation, specialized enzymes carry out minor end processing until the ends are suitable for joining (62). NHEJ is an error-prone pathway as it creates small insertions and deletions (indels) adjacent to the break site.

If extensive end resection has occurred, repair pathway choice is committed to HR as the DNA ends are no longer suitable for ligation. In HR, 5′ to 3′ end nucleolytic processing at the DSB generates a long, single-stranded DNA (ssDNA) overhang. The resected strand conducts a homology search and invades a homologous template, most often the undamaged sister chromatid (63, 64). HR is typically an error-free repair pathway as it leverages a homologous template to restore the original sequence. Due to its reliance on a homologous template, HR is confined to the S and G2 phases of the cell cycle. The extent of DSB end resection dictates pathway choice and commits the repair pathway to either NHEJ, requiring no or minimal resection, or HR, requiring extensive resection (54, 65).

**Figure 4:**
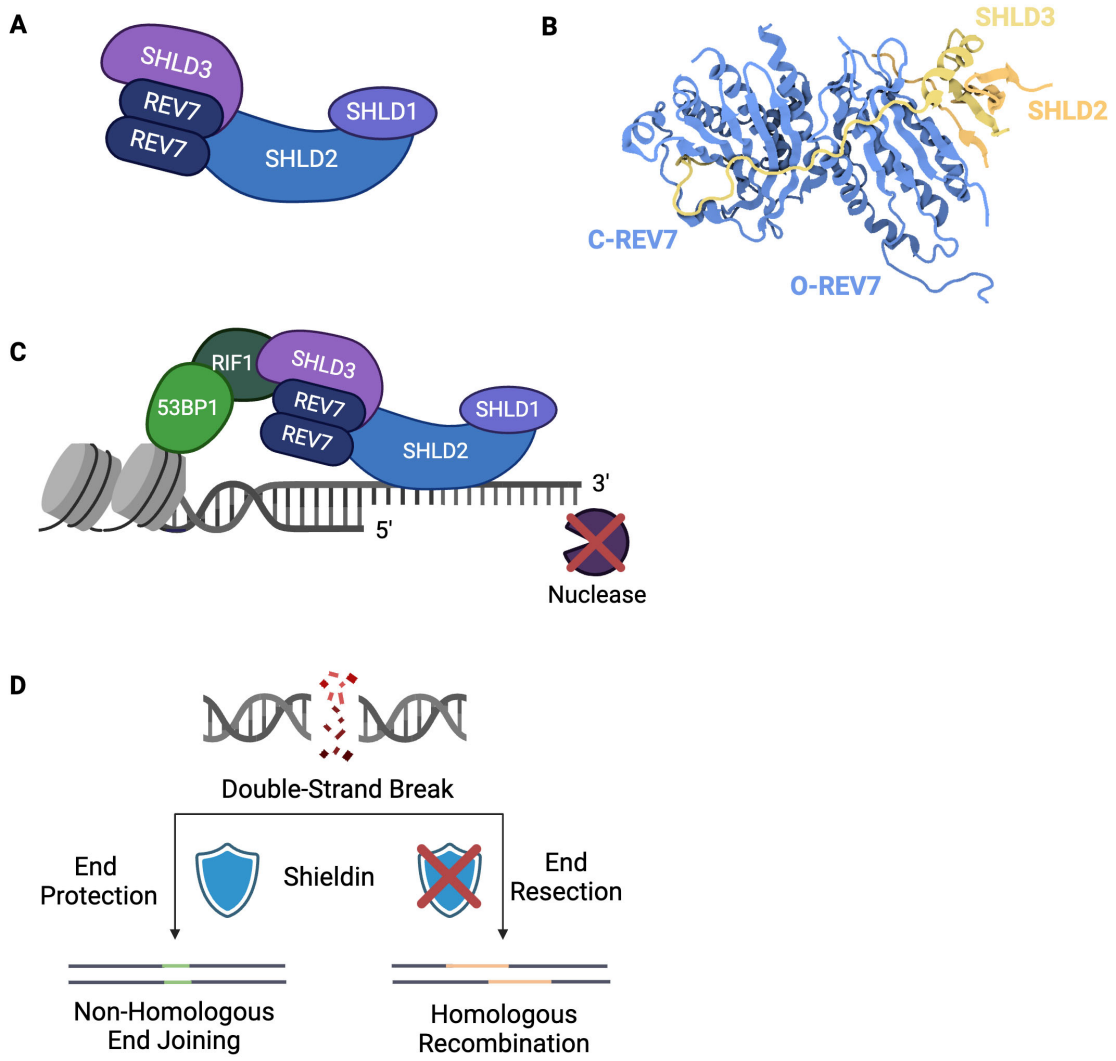
Shieldin Directs Double-Strand Break Repair Pathway Choice. **(A)** The shieldin complex consists of SHLD1, SHLD2, SHLD3, and a REV7 dimer. **(B)** Structure of SHLD3-REV7-REV7-SHLD2 (PDB: 6KTO) (73). In contrast to other REV7-dimer containing complexes, shieldin’s REV7 dimer exists in a unique conformation in which one molecule is in the closed, active state and one molecule is in the inactive, open state. **(C)** Shieldin is recruited to double-strand breaks downstream of 53BP1-RIF1. Upon localization to ssDNA, shieldin protects against 5’ nucleolytic resection. **(D)** Extent of DSB end resection dictates repair pathway choice. Non-homologous end joining requires limited end resection to ensure DNA ends are suitable for direct ligation. Homologous recombination requires extensive end resection. Shieldin blocks DNA end resection, thereby committing repair to non-homologous end joining.

The recent characterization of shieldin has expanded REV7’s various responsibilities to include the regulation of DSB repair pathway choice. This newly characterized complex is composed of a REV7 dimer, SHLD1, SHLD2, and SHLD3 (**Figures 4A, B**) (66–69). Shieldin localizes to DSBs in a 53BP1-RIF-dependent manner and directly binds ssDNA, thereby protecting DNA ends (**Figure 4C**) (66, 67, 70). By preventing further nucleolytic processing, shieldin ensures DSB ends do not undergo extensive resection. Additionally, shieldin recruits a ssDNA binding complex, CTC1-STN1-TEN1 (CST), to DSBs (69, 71). CST interacts with DNA Polymerase alpha/primase (Polα/primase) to conduct fill-in synthesis at the 3’ overhangs as needed (69, 71, 72). Through these roles, shieldin dictates DSB repair pathway choice by blocking HR and promoting repair via NHEJ (**Figure 4D**).

Shieldin relies upon REV7 dimerization for its assembly and activity and, thus, is rendered ineffective if REV7 dimerization is abrogated (68). Unlike other characterized REV7 dimer-containing complexes, in which both REV7 molecules are either in open or closed conformation, shieldin’s REV7 dimer is composed of one open and one closed conformation (**Figure 4B**) (73). One REV7 molecule interacts directly with SHLD3 via its seatbelt domain, taking on a closed, active conformation, whereas the other also interacts with SHLD3 yet remains in an open, inactive state (73). In addition to enhancing the interaction between the REV7 molecules, SHLD3 blocks REV7 from binding REV1, thereby ensuring shieldin does not interact with Pol ζ (73). This likely prevents concurrent use of NHEJ and TLS at the given damage site.

## REV7 contributes to timely mitotic progression

Timely progression through the cell cycle is crucial for faithful genome replication and accurate cell division. Eukaryotic cell division is controlled by a complex network of regulatory mechanisms, ensuring that phase-specific events occur and checkpoints are satisfied before progression to the next phase. This precise regulation guarantees faithful DNA replication, repair of any damage, and proper chromosome segregation. Checkpoint activation delays cell cycle progression until DNA is repaired or chromosomes are properly segregated (74, 75). If DNA damage is irreparable, the checkpoint pathway directs the cell to exit the cycle and may induce cell death. Dysregulation of the cell cycle, a hallmark of cancer, can lead to uncontrolled cell proliferation and the accumulation of additional mutations. REV7 contributes to proper cell cycle progression by inhibiting premature activation of the anaphase-promoting complex/cyclosome (APC/C) and by aiding in mitotic spindle formation.

### REV7 inhibits cell cycle progression by sequestering CDH1

The accurate progression of cells through mitosis is tightly regulated by the APC/C. The APC/C, a multi-subunit E3 ubiquitin ligase, polyubiquitinates key cell cycle regulators, such as cyclins, marking them for proteasomal degradation (76, 77). Sequential activation of the APC/C is mediated by its co-activators, CDC20 and CDH1 (78, 79). The binding of these activators to the APC/C determines which mitotic regulator substrates are targeted for degradation. APC/C^CDC20^ ubiquitinates a limited substrate set, most notably cyclin B and securin, whereas APC/C^CDH1^ ubiquitinates a broader array, facilitating the degradation of remaining mitotic progression regulators (80, 81).

**Figure 5:**
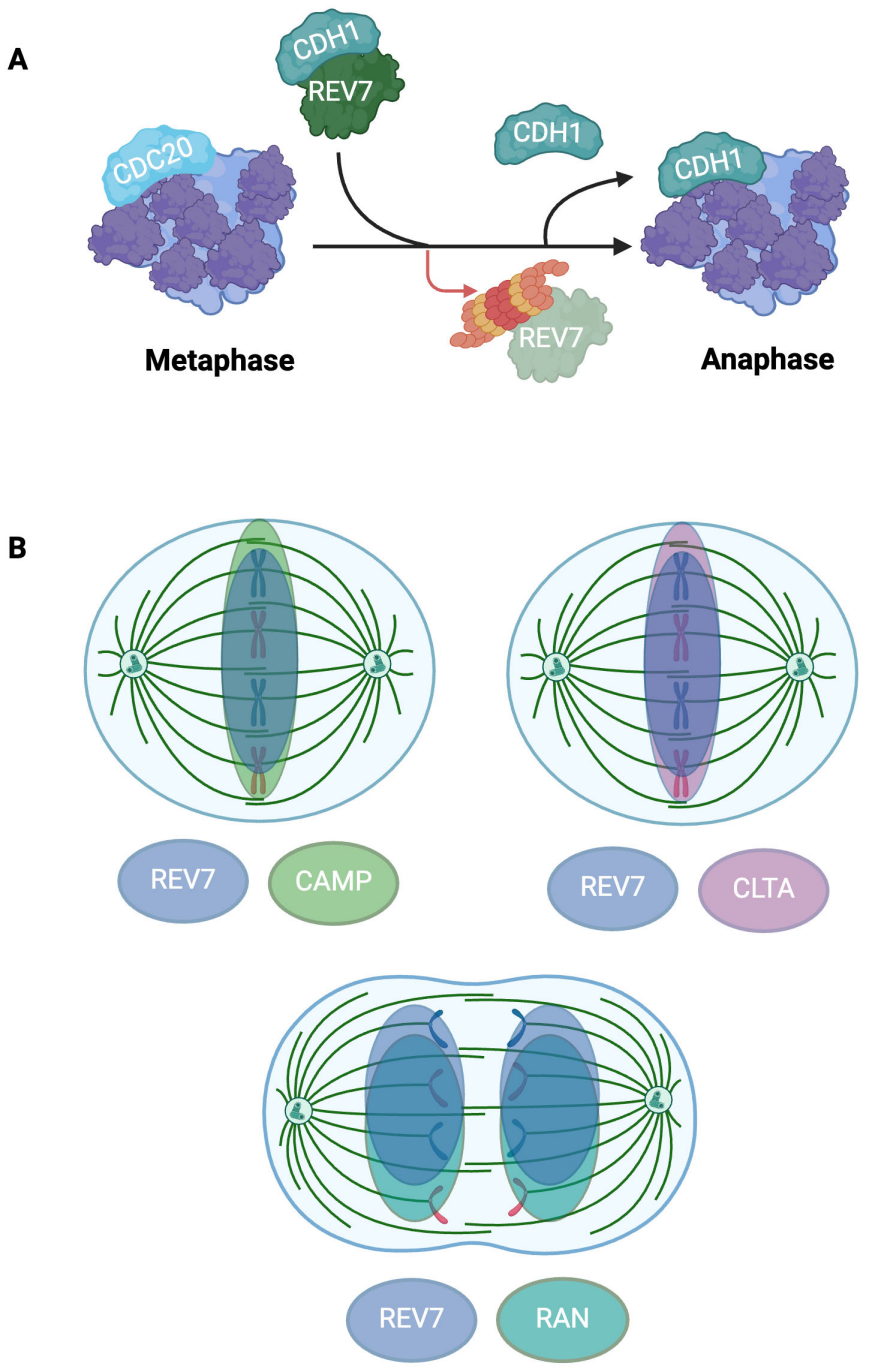
REV7 Modulates APC/C Activation and Co-Localizes To the Mitotic Spindle To Support Mitotic Fidelity. **(A)** REV7 sequesters CDH1 to prevent premature APC/C activation. REV7 sequesters CDH1 during prometaphase and is degraded at the onset of anaphase, thus releasing CDH1 and allowing it to associate with the APC/C. **(B)** REV7 co-localizes with CAMP, CLTA, and RAN at the mitotic spindle. REV7 associates with CAMP and CLTA during metaphase while it interacts with RAN during anaphase. CAMP, CLTA, and RAN are involved with microtubule organization and mitotic spindle fidelity; however, REV7’s exact role remains uncertain.

REV7 prevents premature APC/C activation by interacting with CDH1, thereby regulating the metaphase-to-anaphase transition (5, 82–84). In prometaphase, REV7 sequesters free CDH1 from the APC/C, inhibiting cell cycle progression (**Figure 5A**) (83). In early anaphase, REV7 is swiftly degraded by APC/C^CDC20^, releasing the sequestered CDH1 (**Figure 4A**). The newly released CDH1 then interacts with the APC/C to form the activated APC/C^CDH1^ complex, promoting mitotic progression (**Figure 5A**) (83). Cells depleted of REV7 complete mitosis abnormally quickly, display lagging chromosomes and anaphase bridges, and undergo unscheduled mitotic exit. Notably, REV3-deficient cells do not undergo accelerated mitotic progression or exhibit the same extent of chromosomal abnormalities as REV7-deficient cells, indicating that REV7’s APC/C inhibitory function is independent of Pol ζ (83).

### REV7 co-localizes to the mitotic spindle and protects against spindle assembly checkpoint activation

In addition to its role in inhibiting APC/C^CDH1^, REV7 modulates cell cycle progression by supporting proper mitotic spindle development, thus protecting against spindle assembly checkpoint (SAC) activation (85, 86). The SAC ensures genome stability by delaying cell cycle progression until proper mitotic spindle formation and accurate chromosome segregation have occurred. Misaligned chromosomes and/or distorted mitotic spindles activate the SAC, halting mitotic progression (87, 88). REV7-depleted human cells exhibit spindle abnormalities, aberrant chromosome alignment, and arrest during G2/M (85). Notably, REV3 knockout cells did not undergo cell cycle arrest, indicating REV7’s role in cell cycle arrest is independent of its role in TLS (85). Loss of MAD2 causes mitotic failure and massive cell death due to aberrant spindle assembly, insufficient chromosome condensation, and premature mitotic exit (89). While closely related to MAD2, REV7 loss does not induce mitotic failure or cell death, suggesting that while REV7 is beneficial to accurate mitotic progression, it is not essential. Nonetheless, cells lacking REV7 display MAD2 kinetochore localization, which is indicative of SAC activation, suggesting that REV7 loss activates the SAC (85).

Furthermore, REV7 colocalizes with multiple proteins associated with microtubule organization and chromosome segregation at the mitotic spindle (**Figure 5B**). REV7 directly interacts with chromosome alignment-maintaining phosphoprotein (CAMP), also known as CHAMP1, which is involved in kinetochore-microtubule attachment maintenance (90). The GTPase Ras-associated nuclear protein (RAN), which regulates spindle assembly throughout mitosis, interacts with REV7 throughout the cell cycle (16, 85). Lastly, clathrin light chain A (CLTA), a microtubule stabilizer highly concentrated at the mitotic spindle, interacts with REV7 (91). REV7 depletion causes diffuse dispersal of CLTA throughout the cell and increases the frequency of misaligned chromosomes, highlighting its role in promoting proper progression through mitosis (91).

## REV7 acts in epigenetic reprogramming

Genome instability is intricately linked to developmental defects and infertility (92–96). As such, genomic stability during embryogenesis is imperative for gametic success and propagation of a sexually reproducing species. In mice, REV7 ensures primordial germ cell (PGC) viability, supports reproductive organ development, and is required for fertility. PGCs are the embryonic precursors to oocytes and sperm cells. During mouse PGC development, chromatin undergoes extensive epigenetic remodeling via histone modifications (97, 98). REV7 promotes an open chromatin state in mouse embryonic stem cells and maintains these cells’ pluripotency (99, 100).

### Aberrant primordial germ cell development due to REV7 loss is deleterious to fertility and viability in mice

**Figure 6:**
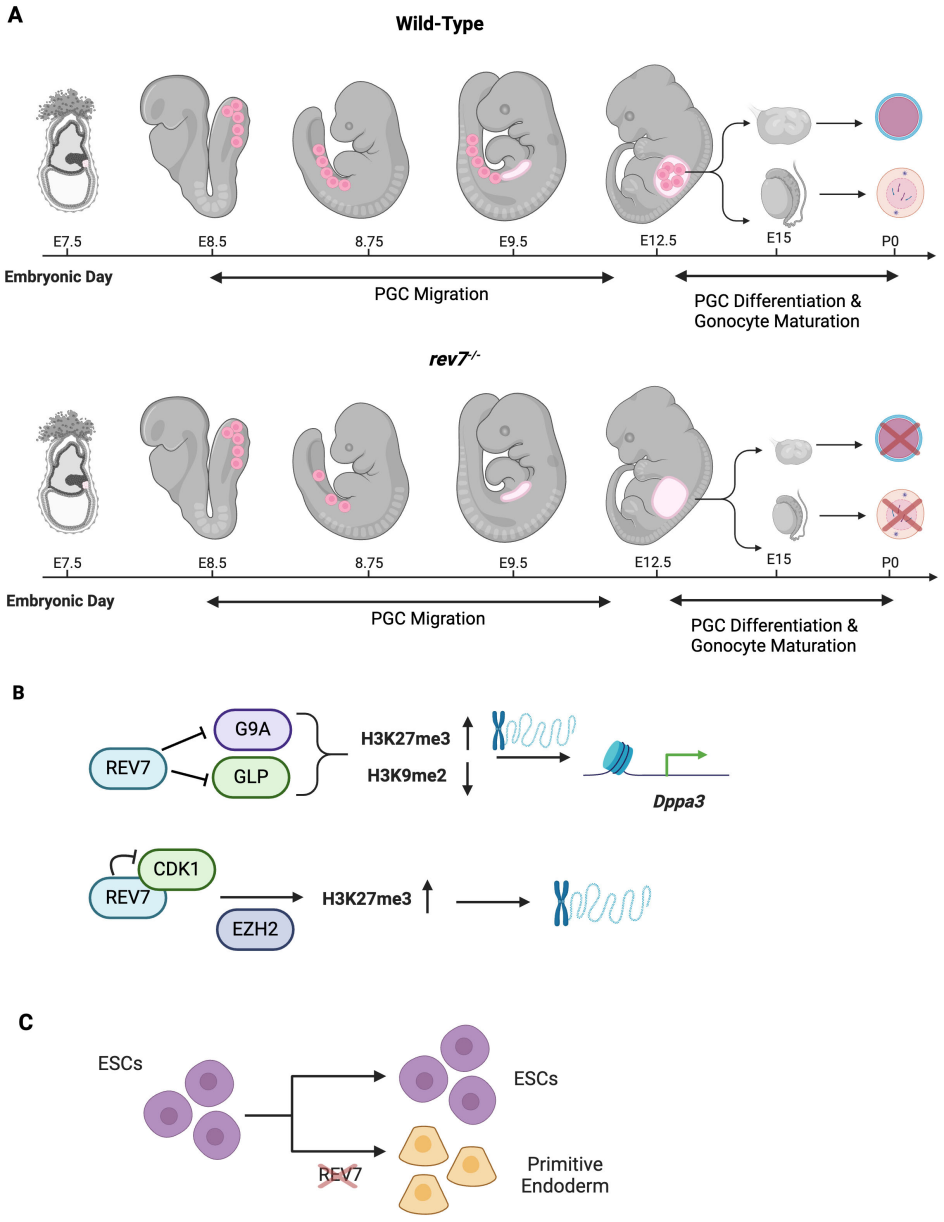
REV7 Promotes Germ Cell Development and an Open Chromatin State. **(A)** REV7 is required for germ cell development and fertility in mice. Primordial germ cells are embryonic precursors to oocytes and sperm cells. In *rev7^-/-^* mice, primordial germ cells (PGCs) are appropriately specified; however, they are lost during migration to the genital ridge. By E9.5, no PGCs remain. Consequently, PGCs do not differentiate into gonads and mice of both sexes are sterile as no primary oocytes or spermatogonial stem cells are produced. **(B)** REV7 promotes an open chromatin state, thus facilitating gene transcription during development. **(C)** REV7 contributes to maintaining ESCs’ pluripotent properties. When REV7 is lost, ESCs spontaneously differentiate into primitive endoderm cells.

During mouse embryogenesis, REV7 is ubiquitously expressed in organs including the brain, lung, colon, kidney, ovary, and testis (101). Postnatally, REV7 expression progressively decreases in all organs but the testes, where it remains highly detectable at P28. REV7-deficient mice of both sexes experience partial embryonic lethality, deviating from Mendelian ratios, with only 10% of progeny being homozygous mutants (102). These mutants exhibit growth retardation, smaller gonads, and most notably, sterility (101–103). The sterility is caused by a complete loss of germ cells in both sexes (**Figure 6A**) (101–103). Male mice lack spermatozoa precursors and spermatozoa while females lack follicles and oocytes, indicating a shared defect in primordial germ cell development (103). Germ cell failure in REV7-deficient mice is attributed to progressive PGC apoptosis as the cells migrate to the genital ridge, indicating REV7 is essential for PGC maintenance (**Figure 6A**) (102).

The development of PGCs in mice requires extensive epigenetic reprogramming (Seki & Saitou, 2005). The primary modifications that mouse PGCs undergo simultaneously are genome-wide DNA demethylation, decreased H3K9 dimethylation (H3K9me2), and increased H3K27 trimethylation (H3K27me3) (97, 104). These modifications occur during PGC migration, temporally aligning widespread epigenetic reprogramming with the progressive loss of PGCs observed in REV7-deficient mice (98). REV7-deficient PGCs fail to decrease H3K9me2 markings and increase H3K27me3 markings, leading to a more repressive chromatin state compared to wild-type PGCs (**Figure 6B**) (105). These findings suggest that the loss of REV7 causes defective epigenetic reprogramming in PGCs due to abnormal methylation patterns.

### REV7 promotes an open chromatin state and maintains pluripotency in mouse embryonic stem cells

Beyond contributing to a less repressive chromatin state in mouse PGCs, REV7 promotes an open chromatin state in embryonic stem cells (ESCs) and maintains the permissive epigenetic marks required for pluripotency. Additionally, REV7 preferentially associates with euchromatin and does not colocalize with H3K9me2-repressively marked heterochromatin (100). Conversely, REV7-depleted ESCs become heterochromatin-enriched and lose pluripotency (100).

*Dppa3*, a maternal effect gene also known as PGC7 or Stella, protects against DNA demethylation processes during early embryogenesis (106). Moreover, DPPA3 is required for the production of fully reprogrammed induced pluripotent stem cells (107). A transcriptome-wide comparison of REV7-deficient ESCs and wild-type ESCs identified significant downregulation of *Dppa3* in REV7-deficient cells (105). Through a sophisticated combination of ChIP-seq, rescue experiments, and DNA methylation analysis, it was shown that REV7 derepresses *Dppa*3 by interacting with the methyltransferases G9a and GLP, thereby decreasing H3K9me2 (**Figure 6B**) (105). Additionally, REV7 can bind cyclin dependent kinase 1 (*Cdk1*), causing G2 arrest and enabling the histone methyltransferase EZH2 to increase H3K27me3, which promotes open chromatin (**Figure 5B**) (100, 105, 108).

REV7 plays a crucial role in maintaining pluripotency in embryonic stem cells (ESCs). Through the promotion of appropriate chromatin structure, REV7 stabilizes factors that counteract ESC instability and differentiation. REV7-deficient ESCs exhibited spontaneous differentiation, even when cultured in leukemia inhibitory factor-supplemented media, which normally maintains pluripotency and prevents differentiation (**Figure 6C**) (107, 109). These cells differentiated into primitive endoderm, accompanied by a reduction in the expression of key pluripotency markers, including Nanog, Oct4, Sox2, and Prdm14 (100). In contrast, GATA binding protein 4, a regulator of cell fate specification, was upregulated due to derepression (110). The maintenance of pluripotency in ESCs relies on an open chromatin state, which is disrupted by repressive epigenetic modifications that favor differentiation (111). Notably, increased H3K9me3 repressive marks, commonly associated with differentiated cells, not ESCs, were observed in the REV7 mutants (100, 112).

## REV7 in immune system development and pathogen response

Further evidence of REV7’s extensive biological activity is demonstrated by its various roles in the mammalian immune system and pathogen response. REV7 is required for antibody diversification, the process by which B cells exploit genome instability pathways to generate a nearly unlimited portfolio of antibodies. Additionally, REV7 is required for B cell viability following cytokine stimulation. In pathogen-specific responses, REV7 can either protect against certain infections or be subverted to propagate illness.

### REV7 promotes high-affinity antibody production

**Figure 7:**
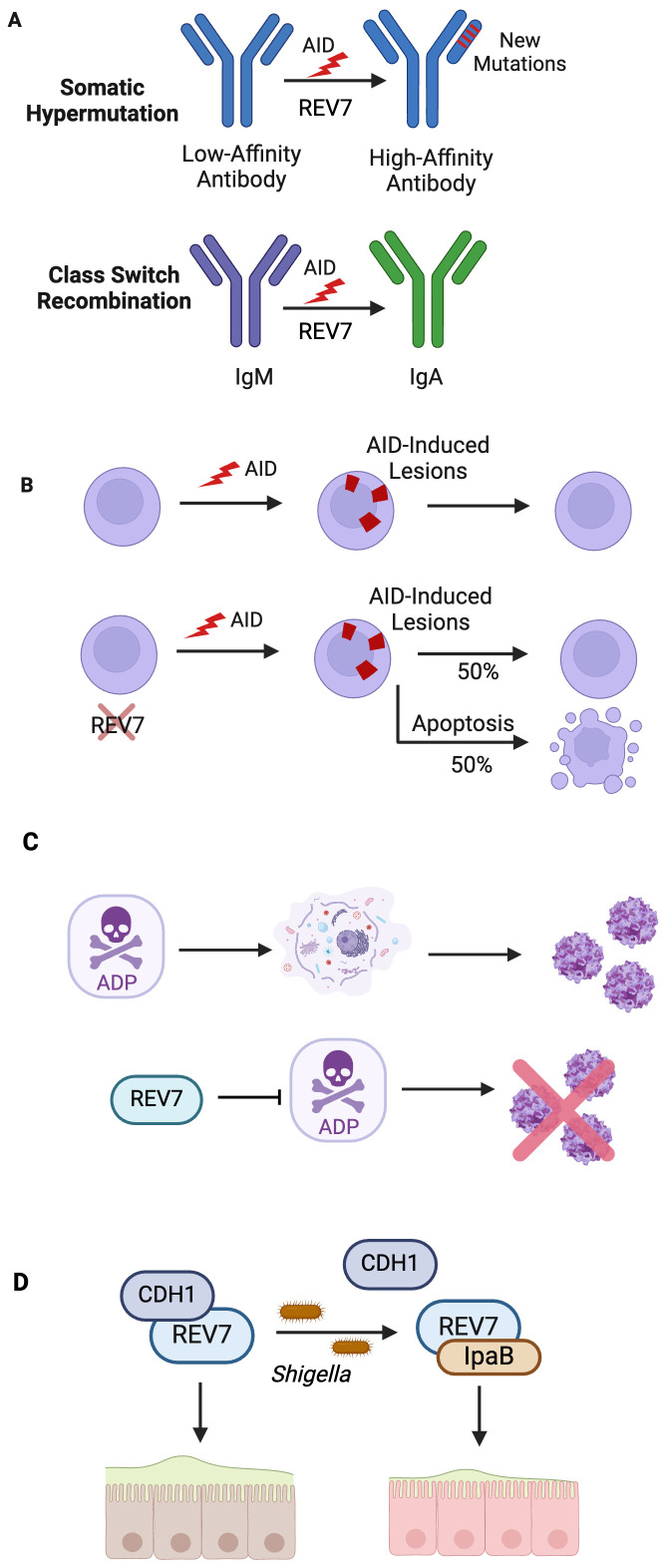
REV7 Bolsters Immunity By Participating in Antibody Diversification and Protecting Against Pathogens. **(A)** SHM and CSR are essential biological processes that generate high affinity, diverse antibodies. In SHM, loss of Pol ζ decreases production of high-affinity antibodies subsequent to low mutation rate. Likewise, loss of shieldin during CSR decreases the rate of productive CSR to 15% of that observed in wild-type B cells. **(B)** REV7 deficiency causes B cell death during CSR. In activated B cells, REV7 is required for AID-induced lesion processing. **(C)** REV7 is involved in pathogen response to *adenovirus* and *shigella*. REV7 protects against further *adenovirus* virion release by binding adenovirus death protein (ADP), which causes cell lysis. **(D)** *Shigella* IpaB co-opts REV7 and disrupts the cell cycle, preventing intestinal epithelial cell renewal, thus promoting bacterial colonization.

B cells are essential to the mammalian immune system, mounting defenses against millions of infectious pathogens. During early B cell development, V(D)J recombination produces a large variety of antigen receptor genes through site-specific recombination of immunoglobulin gene segments (113, 114). The variable (V), diverse (D), and joining (J) gene segments are rearranged and the random combinations produce a highly diverse antibody pool. The recombination event happens through site-specific DSBs, and subsequent repair, at recombination signal sequences that flank each V, D, J gene segment (115, 116). To further expand its pathogen-fighting repertoire, the immune system leverages strategically programmed somatic genome alterations at the IgH locus to produce diverse, high-affinity antibodies (117). These secondary diversification processes are triggered by activation-induced cytosine deaminase (AID), expressed by activated B cells in germinal centers, which deaminates cytosine to uracil (118, 119). This targeted deamination generates mutagenic U:G mismatches and abasic sites, thus initiating both somatic hypermutation (SHM) and immunoglobulin class switch recombination (CSR) (**Figure 7A**) (117, 120).

During SHM, AID-directed mismatches result in untemplated point mutations in immunoglobulin gene regions. AID-induced damage occurs at a rate far exceeding that of spontaneous mutation (121). While base excision repair and mismatch repair pathways repair some abasic sites and gaps, these error-free mechanisms are insufficient to repair the overwhelming volume of damage. Thus, error-prone polymerases, including Pol θ and Pol ζ, are recruited and contribute to Ig hypermutation (122). Pol ζ is constitutively expressed in human B cells but is markedly upregulated during SHM (123). Inhibition of Pol ζ via REV3 inhibition impairs hypermutation, with Pol ζ-depleted B cells exhibiting a 73% reduction in immunoglobulin gene mutation frequency compared to wild type B cells (123).

A study of Pol ζ-depleted mice using antisense RNA to *Rev3* enabled an organism-wide examination of Pol ζ’s role in somatic hypermutation and other immune system-related phenotypes. Despite a significant reduction (30 to 60%) in the number of B cells generated in the bone marrow, these mice mounted robust humoral responses following immunization, including antibody production, isotype switching, and the formation of well-developed germinal centers (124). However, high affinity antibodies, typically generated through SHM, were reduced and the overall mutation frequency decreased. This lower mutation frequency resulted from poorly mutated cells in the Pol ζ-depleted B cell clones, with 62% having three or less mutations compared to 21% of control clones (124). Additionally, the Pol ζ-depleted clones showed a significantly reduced proportion of affinity-enhancing mutations, with only 17% of mutant clones compared to 77% in the control group possessing the necessary alterations. Together, these findings highlight the crucial role that Pol ζ and REV7 itself plays in driving B-cell hypermutation and generating high affinity antibodies.

### Class switch recombination and activated B cell viability necessitate REV7

CSR is the genetic mechanism by which activated B cells switch from synthesizing IgM to producing IgG, IgE, or IgA (**Figure 7A**) (117, 125). Isotype switching enables antibodies to alter their effector functions while retaining their antigen specificity (126). During CSR, AID-induced damage introduces DSBs at specific switch regions throughout the IgH locus, which are subsequently repaired via NHEJ (125, 126). This process is a deletional recombination reaction where opposing S regions recombine, resulting in the loss of the intervening sequence, thereby switching IgM expression to another isotype (127). Shieldin is required for productive CSR as it protects against the resection of AID-induced DSBs, thus permitting repair by NHEJ and alternative end-joining (128). Productive CSR in shieldin-depleted cells occurred at a rate of 15% relative to that observed in wild type B cells, indicating shieldin’s end protection is necessary for productive CSR (128).

REV7-deficient B cells experience approximately a 50% decrease in viability compared to wild-type cells following cytokine stimulation, indicating that REV7 is crucial for B cell survival during CSR (**Figure 7B**) (129). These cells are unable to adequately respond to AID-initiated lesions, with unrepaired abasic sites downstream of these lesions being responsible for the observed cell death. Intriguingly, cells lacking 53BP1 do not exhibit this defect, suggesting that REV7’s role in B cell viability is independent of shieldin (129). Despite REV7’s known function in G2/M arrest, the cell death is due to the inability to adequately repair AID-initiated damage, not aberrant cell cycle progression (129). Cell viability was rescued in AID-deficient cells but not by G2/M arrest. These findings indicate that REV7 is essential to B cell survival during CSR, likely through its role in the damage response to AID-induced lesions via translesion synthesis.

### REV7 protects against prolonged adenovirus infection but stymies the innate immune response to *Shigella*

Adenoviruses are common pathogens that primarily cause upper respiratory tract and gastrointestinal infections. During late infection by species C human adenovirus, adenovirus death protein (ADP) is abundantly expressed and promotes the release of newly synthesized virions by accelerating host cell lysis (130, 131). REV7 was identified as an ADP interactor via yeast two-hybrid screening and validated by human GST pull-downs and co-immunoprecipitations (132). Upon identifying this interaction, lysis studies were carried out in human cells. After adenovirus infection, cell lines overexpressing REV7 lysed less and more slowly than wild-type parental cells. These results indicate that REV7 antagonizes ADP’s cell lytic function, thus curtailing pathogenic virion release (**Figure 7C**). To date, REV7 is the sole known ADP-binding protein (133).

While REV7 is advantageous to the host against adenovirus, *Shigella* uses REV7 against the host. Shigellosis, typified by severe dysentery and colonic inflammation, is caused by the invasion and colonization of colonic epithelial tissue by *Shigella* (134). To promote host cell infiltration, *Shigella* produces invasion plasmid antigen (Ipa) effectors (135, 136). These effectors, many of which dysregulate the eukaryotic cell cycle, enhance pathogen fitness by stimulating bacterial proliferation and/or outwitting the innate immune system (137). A study of *Shigella* infection in HeLa cells and rabbit ileal loops found that IpaB modulates the host’s cell cycle via its interaction with REV7 (138). Upon REV7-IpaB interaction, the APC/C undergoes premature activation, leading to cell cycle arrest at the G2/M phase due to insufficient accumulation of Cyclin B1, Cdc20, and Plk-1 (138). This arrest halts epithelial cell self-renewal, critical to the colon’s innate immune defense against pathogens, thus promoting *Shigella* colonization and survival (**Figure 7D**).

Interestingly, other infectious bacteria have also been shown to utilize REV7 antagonism. Recent studies determined that the *E. coli* effector EspF interacts with REV7 to modulate the host cell cycle, thereby impairing intestinal epithelial cell turnover and promoting prolonged bacterial colonization (139).

## DNA damage response & cancer development

The predominant hallmarks of cancer include genome instability, increased mutagenesis, resistance to apoptosis, and abnormal proliferation, which may occur through sustained proliferative signaling or evasion of growth suppressors (140). These cancer-promoting characteristics can result from dysregulation of underlying molecular pathways, such as those involved in DDR and cell cycle progression. As previously discussed, REV7 functions in the DDR as a member of Pol ζ and shieldin, and also acts as an APC/C modulator to ensure proper cell cycle progression. Consequently, it has significant implications for carcinogenesis, disease progression, patient outcomes, and the development of chemoresistance.

The goal of chemotherapy and radiation therapy is to inhibit cancer cell proliferation, thereby preventing tumor growth and metastasis. Traditional chemotherapy and radiation therapy induce DNA damage in cancer cells, leading to cell death either directly or through the activation of apoptotic pathways (141, 142). Upon DNA damage recognition, the DDR is activated, which leads to repair, lesion tolerance, or triggers cellular senescence or apoptosis. However, these responses, which are typically beneficial, can become co-opted or dysregulated, thus contributing to genome instability and carcinogenesis. For instance, the REV1-Pol ζ mutasome can directly replicate both spontaneous lesions and cisplatin-induced lesions. While this replication prevents fork stalling and collapse in the case of spontaneous lesions, when it occurs at cisplatin-induced lesions, it allows the cell to avoid chemotherapy-induced apoptosis, ultimately undermining the treatment’s efficacy.

All cancer cells carry somatic mutations and their genomes evolve from once-normal cells through a series of acquired mutational events (143). These mutations provide cancer cells with advantageous traits, such as sustained proliferation, evasion of apoptosis, epithelial-to-mesenchymal transition, and resistance to genotoxic stress. These cells undergo positive selection, driving tumor initiation, disease progression, and chemotherapeutic resistance. This becomes particularly perilous when genes encoding DDR factors are mutated in cancer, thereby exacerbating genome instability. These mutations allow cancer cells to evade protective DDR pathways, increasing error rates and amplifying the mutational burden, which heightens the likelihood of oncogene activation and tumor suppressor gene inactivation.

## Translesion synthesis & chemotherapeutic resistance

Xeroderma pigmentosum (XP), a rare hereditary skin syndrome characterized by extreme photosensitivity and a predisposition to skin cancer, was the first condition to definitively link defective TLS with carcinogenesis. This disorder is caused by mutations in XP complementation group genes, all of which are involved in repairing or tolerating UV-induced damage (144–146). XP-V is caused by mutations in *PolH*, a gene encoding a specialized TLS polymerase used for synthesis past UV-induced cyclobutane pyrimidine dimers, leading to sun exposure-induced carcinogenesis (147, 148). Unlike the upcoming discussion, where TLS contributes to cancer initiation and progression, the absence of TLS is carcinogenic in XP. Nevertheless, the link between XP and TLS is crucial, as it was the first to connect dysregulated TLS to cancer, spurring much of the subsequent research on this relationship.

### Mutagenic TLS contributes to genome instability that promotes malignancy

**Figure 8:**
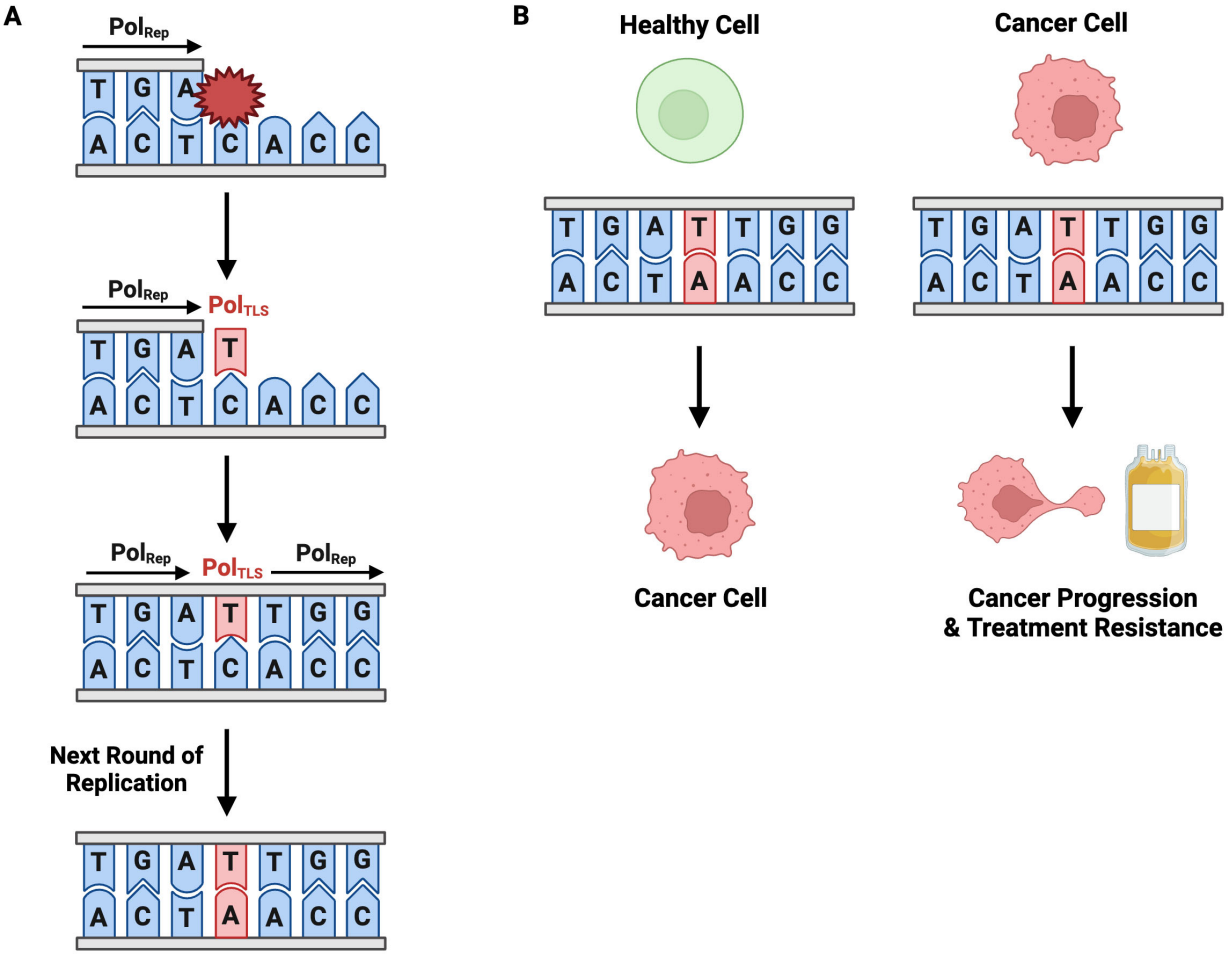
Mutagenic TLS Contributes to Carcinogenesis, Disease Progression, and Treatment Resistance. **(A)** TLS is an inherently mutagenic pathway. Incorporation of an erroneous nucleotide during TLS is permanently codified in the genome through subsequent rounds of replication. **(B)** TLS-induced point mutagenesis contributes to cancer development in healthy cells and cancer-progression and treatment resistance in cancerous cells.

TLS is an error-prone pathway, making it inherently mutagenic. High-fidelity replicative polymerases synthesize DNA with a nucleotide misincorporation rate of one in 10^-6^ to 10^-8^, whereas TLS polymerases have misincorporation rates ranging from 10^-1^ to 10^-4^ (148). Pol ζ, the only B-family translesion polymerase, has a single base substitution rate of approximately 10^-3^. This significantly higher misincorporation rate is due to a combination of permissive active sites and a lack of exonuclease proofreading activity (27). While TLS plays an invaluable role in lesion bypass and ensuring replication fork progression, its tendency to introduce mutations can drive the genome instability that underlies carcinogenesis (**Figure 8A**). This becomes particularly problematic when TLS is over utilized relative to high-fidelity polymerases. TLS-induced mutations can initiate or promote cancer development. For instance, a mutation could transform a healthy cell into one with malignant properties by inactivating a tumor suppressor gene, activating an oncogene, or conferring malignant growth properties (e.g., sustained proliferation, evasion of apoptosis) (**Figure 8B**). In the context of cancer, TLS-induced mutagenesis can lead to the creation of heterogeneous, therapy-resistant clones, which are subsequently selected during clonal expansion. This facilitates tumor evolution and may render treatment ineffective.

### TLS allows cells to evade genotoxic therapy-induced apoptosis by tolerating damage

Chemotherapy and radiation therapy exert anti-neoplastic activity by directly or indirectly damaging DNA with the aim of inducing cancer cell death. A treatment’s cytotoxicity and consequent success are proportional to the lesions it induces stalling replication fork progression and causing cell death. Intrinsic and acquired therapeutic resistance allow cancerous cells to evade cell death despite these lesions and continue proliferating, thus driving tumor progression (149, 150). TLS is a crucial contributor to both intrinsic and acquired resistance. Intrinsic and acquired resistance can be attributed to TLS’ insertion step and extension step, respectively. The nucleotide insertion across from the lesion is intrinsically treatment-resistant as it allows direct replication of therapy-induced DNA damage, which would otherwise block a standard replicative polymerase. The extension step, in which a second polymerase (e.g., Pol ζ) extends from the damaged termini, is highly mutagenic and could introduce mutations that promote cancer and lead to acquired chemotherapeutic resistance (**Figures 8A, B**).

Cisplatin, a platinum-based chemotherapeutic, is the first-line treatment for patients diagnosed with a wide spectrum of solid tumors including lung, breast, ovarian, testicular, and bladder cancers (151). Likewise, it is the predominant treatment for hematological malignancies like leukemia and multiple myeloma (151). Cisplatin exerts its cytotoxicity by introducing intrastrand and interstrand crosslinks and monoadducts (152). These sterically bulky lesions block synthesis and distort the DNA helix, causing replication arrest and cell death (153, 154). Pol ζ is efficient in TLS across cisPt-GG adducts, the most common cisplatin-induced lesion (152). As such, Pol ζ is used in adduct bypass in more than 80% of cisPt-GG adducts in mammalian cells (155). Despite its prominent use in this type of lesion bypass, Pol ζ synthesis is at least 7-fold more mutagenic than other TLS polymerases at cisPt-GG adducts (155). Pol ζ is also involved in the bypass of radiation therapy-induced thymine glycol (Tg) lesions, albeit with higher fidelity than the bypass of other lesions (156).

Beyond its role in tolerating cancer treatment-induced damage like cisPt-GG adducts and Tg lesions, Pol ζ is proficient in TLS extension in human cells when challenged with abasic sites, benzo[SB ITAL *a*]pyrene-guanine (BP-G) and 4-hydroxyequilenin-C (4-OHEN-C) lesions, and thymine-thymine 6-4 photoproducts (TT 6-4 PP) (155). In line with its mutagenicity in extending cisPt-GG adducts, Pol ζ misinsertion frequency is 74% and 75% at 4-OHEN-C and TT 6-4 PP lesions, respectively (155). These elevated mutagenesis rates contribute to overall mutational burden, which may include mutations associated with cancer hallmarks and acquired chemoresistance. Taken together, these findings highlight the significant role of Pol ζ in carcinogenesis and treatment resistance.

## REV7 overexpression is associated with carcinogenesis & treatment resistance

High levels of REV7 expression are observed in various cancers including glioma, ovarian, cervical, testicular, pancreatic, breast, and lung. REV7 overexpression is linked to pro-malignant traits such as hyperproliferation and epithelial-mesenchymal transition (EMT). In many of these cancers, high REV7 expression is correlated with chemotherapeutic resistance and radioresistance, thus reducing survival (**Table 3**) (157–162). At wild-type baseline expression, REV7’s role in Pol ζ renders it highly mutagenic. Therefore, REV7 overexpression in cancerous tissue, particularly during disease progression, is a logical outcome based on REV7’s inherent increased mutagenic propensity. Likewise, overexpression of REV7 may allow Pol ζ to outcompete other polymerases and bypass cisPt-GG adducts and Tg lesions. Studies on REV7 overexpression in colorectal cancer show conflicting results, with some suggesting that high expression is associated with a decreased risk, while others indicate it contributes to treatment resistance (163–165). In cervical, pancreatic, nasopharyngeal, glioma, testicular, and lung cancer cell lines, REV7 depletion increases sensitivity to chemotherapy or radiotherapy (**Table 4**) (160–162, 166–175). These data suggest REV7 expression is tightly regulated in normal cells due to its mutagenic potential but becomes deregulated in cancerous cells, further augmenting Pol ζ’s contributions to carcinogenesis and treatment resistance.

**Table 3:** REV7 predicts outcomes In various cancers.

Cancer Type	Prognostic Indicator	Reference
Triple negative breast	High REV7 expression is associated with shorter overall survival	(157)
Diffuse large B-cell lymphoma	High REV7 expression is associated with shorter progression-free survival and overall survival in patients treated with rituximab	(158)
Gastric	High REV7 expression is associated with shorter overall survival	(157)
Lung	High REV7 expression is associated with shorter overall survival	(157)
Melanoma	High REV7 expression is associated with increased tumor thickness	(159)
Ovarian	High REV7 expression is associated with shorter overall survival	(182)
Ovarian clear cell carcinoma	High REV7 expression is associated with shorter progression-free survival in advanced disease	(181)
Pancreatic ductal adenocarcinoma	High REV7 expression is associated with shorter overall survival in unresectable PDAC treated with platinum-based chemotherapy	(160)
Testicular germ cell	High REV7 expression is associated with chemotherapeutic resistance and shorter overall survival	(161, 162)

**Table 4:** Consequences of REV7 depletion in cancer studies.

Organ	Cancer Type	REV7 Depletion	Reference
Bladder	Bladder	Inhibits cancer cell proliferation and viability	(173)
Breast	Triple negative	Inhibits breast cancer cell migration, invasion, and EMT	(174)
Cervical	Cervical	Enhances sensitivity to cisplatin	(166)
Esophagus	Esophageal squamous cell	Increases radiosensitivity; decreases tumor burden in mice	(175)
Brain	Glioma	Inhibits cell proliferation; increases sensitivity to cisplatin	(170)
		Increases ionizing radiation-induced cytotoxicity; increases rate of damage due to ionizing radiation	(168)
		Increases radiosensitivity; inhibits CD8+ cell death	(169)
Lung	Small cell	Suppresses cell proliferation; activates apoptotic pathway	(172)
	Non-small cell	Increases sensitivity to cisplatin and doxorubicin	(252)
		Promotes cisplatin sensitivity; increases cisplatin-induced senescence	(171)
		Inhibits cell migration, invasion and EMT; decreases distant metastasis	(211)
Pharynx	Nasopharyngeal	Increases sensitivity to cisplatin and irradiation; decreases rate of mutagenesis	(167)
Ovary	Ovarian clear cell carcinoma	Decreases cell proliferation; increases sensitivity to cisplatin	(181)
Pancreas	Pancreatic ductal adenocarcinoma	Reduces proliferation; increases sensitivity to cisplatin	(160)
Skin	Melanoma	Reduces melanoma cell proliferation, migration, and invasion	(159)
Testicle	Testicular germ cell	Increases sensitivity to cisplatin and doxorubicin; overcomes chemoresistance; decreases cell proliferation	(161, 162)

### Ovarian cancer

Ovarian cancer (OC) is a gynecological malignancy with a 51% 5-year relative survival rate, affecting approximately 20,000 women annually in the United States (176). Ovarian clear cell carcinoma (OCCC), a histotype of OC, comprises approximately 11% of annual cases (177). Among OC histotypes, OCCC is associated with poor outcomes due to chemotherapeutic resistance and disease recurrence (178–180). In women receiving platinum-based chemotherapy after surgery, only 11% of OCCC patients responded to chemotherapy, compared to 72% of serous carcinoma patients (178). REV7 is frequently expressed in OC, with 92% of patient-derived tissue positive for REV7 expression (181). This correlation is particularly pronounced in OCCC, where 100% of samples were REV7-positive. Additionally, higher REV7 is associated with poorer prognosis, as measured by progression-free survival. Further investigation in REV7-knockdown OCCC cells and a mouse tumor xenograft model solidified the importance of REV7 in OCCC and its critical contribution to chemotherapeutic resistance.

OCCC cells depleted of REV7 via RNAi showed suppressed proliferation without affecting cell cycle progression, indicating that decreased proliferation was due to REV7 loss, not cell cycle arrest (181). When treated with cisplatin, REV7 knockdown cells responded positively to treatment, as evidenced by elevated DSBs and increased apoptosis in a dose-dependent manner. These findings were reinforced in a mouse model, where REV7-depleted OCCC tumors grew more slowly than control OCCC tumors. Moreover, the mice bearing REV7-depleted tumors responded to cisplatin and showed a significant decrease in tumor volume compared to cisplatin-treated control OCCC.

A recent study identified novel tumor-promoting biochemical roles for REV7, indicating its carcinogenic properties extend beyond those associated with the DDR. Specifically, it implicated REV7 in inflammatory responses, increased cell proliferation and migration, and the inhibition of ferroptosis (182). Tumor-infiltrating CD4, CD8, NK, macrophage, and dendritic cells were positively correlated with REV7 expression. Increased REV7 expression also accelerated cell proliferation, migration, and invasion in a human OC cell line. Notably, REV7 overexpression inhibits ferroptosis, an iron-mediated form of programmed cell death characterized by the excessive accumulation of lipid peroxides on cellular membranes (183). As ferroptosis acts as a natural tumor suppressor, this reveals another pro-carcinogenic function of REV7 (184).

### Glioma

Gliomas are a heterogeneous collection of primary brain tumors classified by histomorphological characteristics and established central nervous system tumor biomarkers (185, 186). These tumors develop from aberrant epigenetic reprogramming and/or mutations in single brain cells (185). While prognosis is good in low-grade gliomas, the vast majority of gliomas are high-grade. High-grade gliomas, known as glioblastomas (GBM), are aggressive and associated with very poor outcomes due to treatment resistance and failure. Factors including the inability to resect or poor tumor resection, chromosomal instability, mutated DDR genes, permissive epigenetic states, and stem-like cells, make glioblastomas particularly perilous malignancies (185, 187–190). Accordingly, glioblastoma standard-of-care is aggressive and multi-modal, employing a combination of therapeutic approaches including surgical resection, radiation therapy, and chemotherapy (191). Nonetheless, resistance to ionizing radiation rapidly develops and is nearly inevitable. A unique and major contributor to treatment failure in these tumors are intrinsically radiation-resistant glioblastoma stem-like cells, which exhibit DDR hyperactivation (192). As such, sensitizing glioblastomas to radiation therapy would be distinctly advantageous due to current standard-of-care limitations.

REV7 is overexpressed in both low-grade glioma tissue and GBM tissue, exhibiting an average of 4-fold increase over adjacent tissue (168–170). Likewise, multiple glioma and glioblastoma cell lines show a marked increase in REV7 expression, ranging from a 2.5-fold increase to a 4-fold increase compared to expression levels in normal astrocytes (169). In patients, high REV7 expression level is associated with increased tumor size (170). REV7 loss sensitizes glioma cells to ionizing radiation by reducing cell proliferation and increasing cell death (168, 193). Sensitization, at least in part, is attributed to elevated radiation-induced DNA damage as evidenced by increased γ-H2AX, a marker of DSBs (168, 193). Moreover, REV7 depletion inhibits the PI3K/AKT/mTOR pathway, a signaling pathway that when aberrantly activated is oncogenic and contributes to radiation therapy resistance (169, 170, 194). In addition to sensitizing glioma cells to radiation therapy, REV7 depletion also sensitizes glioma cells to cisplatin treatment (170).

While the negative outcomes associated with high REV7 expression in glioma are certainly partially attributable to Pol ζ-driven mutagenesis, data suggest that aberrant REV7 expression is carcinogenic through an additional mechanism unrelated to mutagenic TLS. Studies comparing the expression of REV3, Pol ζ’s catalytic subunit, and REV7 in gliomas show variation between pathophysiological and clinical characteristics. In GBM, REV7 expression is significantly increased in cancerous tissue compared to adjacent paracancerous tissue, whereas no statistical difference exists between REV3 expression in diseased tissue and paracancerous tissue (170). Furthermore, a greater proportion of REV7 depleted GBM cells undergo apoptosis than REV3 depleted GBM cells upon radiation (169). Finally, high REV7 expression is correlated with a more rapid decline in patient survival than high REV3 expression (169).

### Breast cancer

Breast cancer is the most prevalent cancer among women, with an estimated lifetime incidence of 1 in 8 (195). As a highly heterogeneous disease, it is classified into subtypes based on immunohistochemical and molecular characteristics. Increased breast cancer screening, enabling early detection, and improved therapeutic strategies have led to a 5-year relative survival rate of 91%, making breast cancer one of the most survivable malignancies (195). However, this remarkable survival rate is not uniform across all subtypes. Triple-negative breast cancer (TNBC) is a particularly aggressive subtype of invasive breast cancer characterized by the absence of estrogen receptors (ER), progesterone receptors (PR), and human epidermal growth factor receptor 2 (HER2) overexpression (196, 197). Although TNBC accounts for approximately 13% of all breast cancers, it disproportionately contributes to breast cancer mortality, responsible for 40% of all related deaths (198). This elevated mortality rate is attributed to a high risk of recurrence, increased likelihood of metastasis, and limited treatment options. Women with TNBC are more likely than those with non-TNBC to develop metastases in the brain and lungs, with rates of 30% versus 10% and 40% versus 20%, respectively (199–201). Due to the absence of ER, PR, and HER2, TNBC does not respond to standard breast cancer endocrine therapies (e.g., tamoxifen, aromatase inhibitors) or trastuzumab, a monoclonal antibody targeting HER2.

REV7 is highly expressed in resected breast cancer tissue and increased expression is associated with decreased survival rates (157, 174). Breast cancer cells from distant metastatic tissue had the highest REV7 expression (174). Likewise, REV7 is overexpressed in various breast cancer cell lines with the most significant increase noted in TNBC cells (157, 174). REV7 knockdown impairs the migratory, invasive, and pro-EMT capacities of TNBC cells, thereby limiting their metastatic potential (174). Concordantly, REV7 overexpression promotes EMT. Subsequent studies revealed that TNBC cells overexpressing REV7 exhibit mitotic dysregulation, characterized by a failure to complete mitosis, abnormally slow cell cycle progression, mitotic slippage, and aberrant stabilization of APC/C substrates following nocodazole release (157). Taken together, these findings suggest that REV7 plays a critical role in breast cancer cell growth, migration, invasion, and epithelial-mesenchymal transition, all of which contribute to disease progression, particularly distant metastasis, and poor clinical outcomes.

### Skin cancer

Ultraviolet (UV) radiation is a major carcinogen and a primary risk factor for skin cancer development, closely associated with the initiation of basal cell carcinoma (BCC), squamous cell carcinoma (SCC), and malignant melanoma (MM) (202–204). UV radiation exposure induces various forms of DNA damage that promote cancer-driving mutations, facilitating carcinogenesis and disease progression. The most common DNA lesions caused by UV radiation are cyclobutane pyrimidine dimers, followed by 6–4 photoproducts and 8-oxo-guanines (205). Through a multistep process involving cycles of unrepaired photoproducts, mutagenesis, and clonal selection, these lesions contribute to the initiation, promotion, and progression of skin cancers (206). Nucleotide excision repair (NER) is the primary mechanism employed by cells to repair these lesions; however, TLS is also utilized. Notably, Pol ζ, of which REV7 is a subunit, commonly serves as the extension polymerase in mutagenic two-step TLS during UV photoproduct bypass (27).

A study examining REV7 expression in skin cancers revealed elevated levels in BCC, SCC, and MM, with 83%, 75%, and 91% of tissues exhibiting REV7 expression, respectively (159). In MM, REV7-positive samples were classified into low and high intensity expression groups, with high-intensity expression correlating strongly with increased cell proliferation and tumor growth (159). Moreover, high intensity REV7 expression was associated with greater tumor thickness, a critical prognostic marker, underscoring REV7’s role in MM disease progression (207). Functional studies in MM cell lines demonstrated that REV7 depletion inhibits cell proliferation, motility, and invasion, all key factors of disease progression. Furthermore, REV7 loss sensitized MM cells to cisplatin treatment (159).

### Lung cancer

Lung cancer is the leading cause of cancer-related deaths in the United States, claiming 125,000 lives annually—more than any other form of cancer (208). In addition to being an inherently aggressive malignancy, acquired chemoresistance due to dysregulated DDR processes is a significant contributor to lung cancer’s high mortality rate (209, 210). Elevated levels of REV7 have been detected in patient-derived non-small cell cancer (NSCLC) and small cell lung cancer (SCLC) samples, as well as in various lung cancer cell lines and mouse models (157, 172, 211). High REV7 expression is correlated with metastatic disease and decreased survival (211). In lung cancer cells, REV7 overexpression is associated with not only increased cell proliferation but also with enhanced cell migration, invasion, and EMT, all of which are oncogenic processes and promote disease progression (211). Correspondingly, REV7 depletion suppresses these processes (211). Recent gene expression analyses in REV7-overexpressing cells have identified slug, a transcription factor that promotes EMT, as highly enriched (211, 212). This finding is consistent with the theory that REV7 promotes EMT, at least in part, through the regulation of slug.

While lung cancer often initially responds well to platinum-based chemotherapeutics like cisplatin, treatment resistance remains a major consideration and contributes to lung cancer’s high mortality rate (213). In lung cancer, REV7 knockout sensitized cells to cisplatin and re-expression of wild-type REV7 in these cells restored cisplatin-resistance (171). Likewise, REV7 loss in a cisplatin-resistant NSCLC mouse model increased sensitivity to cisplatin as evidenced by decreased rate of tumor volume growth and increased survival, with the *REV7* mutants living twice as long as the wild-type mice (171). Consistent with the findings in gliomas, REV7 loss promotes cisplatin sensitivity of lung cancers more potently than REV3 loss, further supporting REV7’s chemosensitizing properties beyond its role in Pol ζ. Interestingly, cisplatin sensitivity and associated improved outcomes are attributed to cancer cell senescence, rather than apoptosis. After cisplatin treatment, REV7 depleted cells showed decreased levels of cleaved caspase 3, an apoptotic cell death marker, indicating the loss of cell viability occurs in an apoptosis-independent mechanism. The REV7 depleted cells become senescent upon cisplatin treatment, as evidenced by their flattened morphology, a physical hallmark of senescent cells, and beta-galactosidase activity initiation (214).

Moreover, specific single-nucleotide polymorphisms (SNPs) in REV7 are associated with chemotherapy response and prognosis in lung cancer patients (215, 216). A significant correlation exists between the REV7 rs746218 polymorphism and progression-free survival in patients treated with cisplatin (216). Lung cancer patients with the AG or AA genotype exhibited notably longer progression free survival compared to those with the GG genotype. As REV7 rs746218 is a variant in the promoter region, its effect may arise from modulating REV7 gene expression (216).

## Small molecule inhibition of REV1-Pol ζ sensitizes multiple cancers to cisplatin and reduces mutagenesis

Mutagenic TLS plays a critical role in the development of chemoresistance, as this pathway mediates both intrinsic and acquired chemoresistance (217). Consequently, TLS polymerase inhibition is a promising adjuvant target for enhancing chemotherapeutic efficacy. While platinum-based chemotherapeutics like cisplatin are initially highly potent cytotoxic agents, their efficacy is constrained by the swift development of resistant clones following repeated exposure to the chemotherapeutic agent (152). A treatment regimen combining a front-line chemotherapeutic like cisplatin with a TLS inhibitor would not only increase cancer cell chemosensitivity but also decrease chemotherapeutic resistance, reducing cancer recurrence and secondary malignancies, therefore improving patient outcomes.

**Figure 9:**
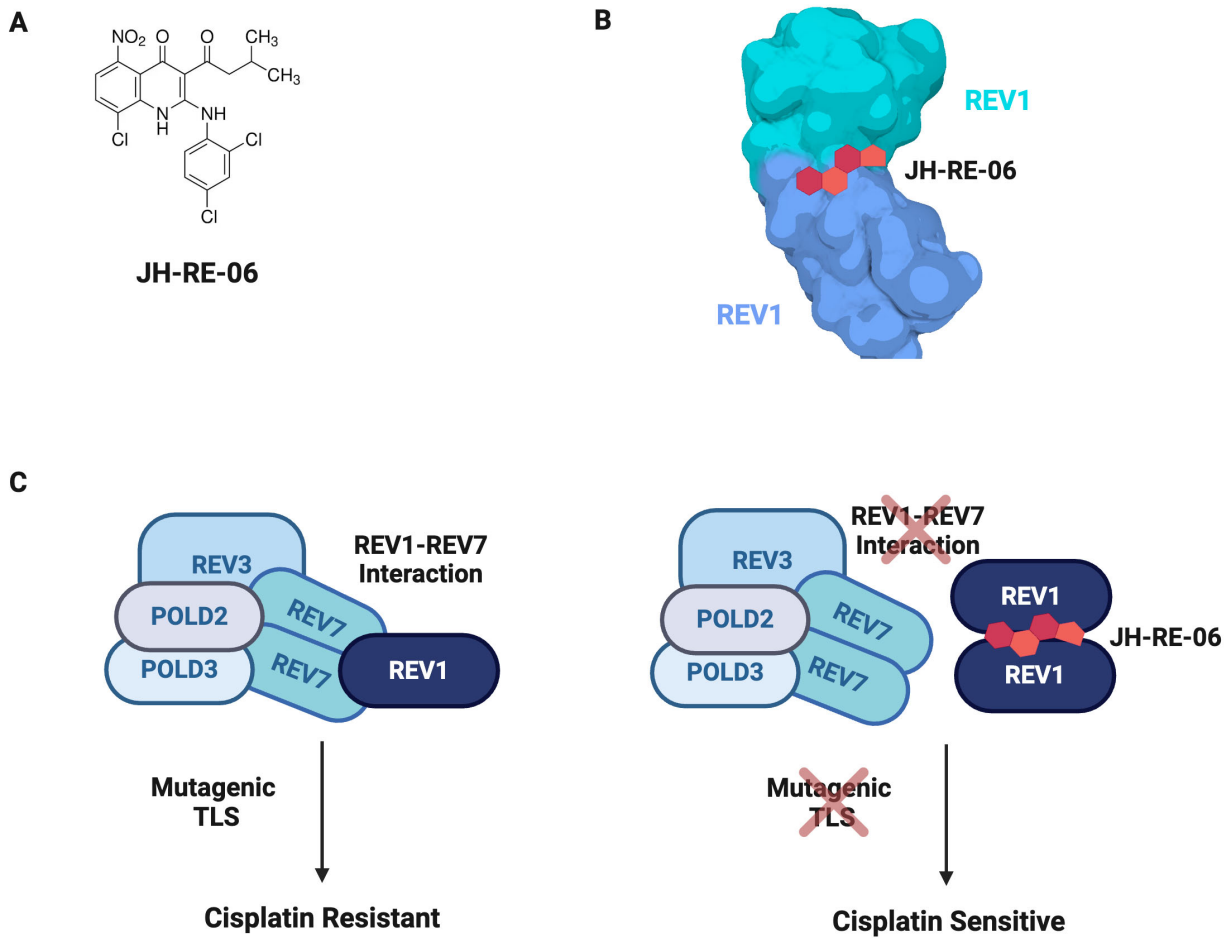
JH-RE-06 Is A Potent TLS Inhibitor. **(A)** JH-RE-06 is a small molecule inhibitor of mutagenic TLS by the REV1-Pol ζ mutasome. **(B)** JH-RE-06 binds REV1 and induces dimerization. When REV1 is dimerized, REV7 cannot bind REV1. **(C)** JH-RE-06 sensitizes cancer cells to cisplatin. JH-RE-06 blocks the interaction between REV1 and REV7, preventing assembly of the REV1-Pol ζ mutasome. As TLS is suppressed, cisplatin resistance due to mutations acquired during error-prone TLS is avoided.

A small molecule screen of compounds able to bind REV1 identified JH-RE-06 as a potential TLS inhibitor (**Figure 9A**) (218). JH-RE-06 binds REV1 and promotes dimerization at the REV7-binding surface, therefore disrupting the REV1-REV7 interaction (**Figure 9B**) (218). As the REV1-REV7 interaction is required for Pol ζ-extended TLS, blocking this interaction abrogates mutagenic TLS (**Figure 9C**). A particularly compelling characteristic of JH-RE-06 is the surface it interacts with is vital to error-prone TLS but not high fidelity lesion bypass, thus restricting its action to only mutagenic lesion bypass (218). In multiple cancer cell lines, JH-RE-06 sensitizes cells to cisplatin, indicated by decreased cell survival, as well as inhibits cisplatin-induced mutagenesis, thus stymieing acquired chemoresistance. In a mouse melanoma model, administration of saline, cisplatin, or JH-RE-06 resulted in the same rate of tumor volume growth; however, co-administration of cisplatin and JH-RE-06 strikingly showed nearly no tumor volume growth over 30 days. Likewise, mice administered saline, cisplatin, or JH-RE-06 died at a similar time point, whereas mice treated with both cisplatin and JH-RE-06 outlived those groups by nearly 50%. Subsequent work, both *in vivo* and *in vitro*, established JH-RE-06 alters cell response to cisplatin by inducing senescence and repressing apoptosis (219). While a TLS inhibitor is promising adjuvant therapy, further work must elucidate whether a TLS inhibitor would act preferentially on cancer cells and whether TLS loss is cytotoxic to normal cells. Furthermore, it will be imperative to conduct studies to determine whether the loss of mutagenic TLS by REV1 inhibition activates compensatory mechanisms, potentially driving disease progression and treatment resistance.

## Concluding remarks & remaining questions

Since its discovery forty years ago, REV7 has often been dismissed as an “accessory” protein despite acting in crucial processes including cell cycle regulation and TLS. However, research in the past several years has firmly established REV7 as a *bona fide* genome stability regulatory protein. The exciting discovery of the shieldin complex established that REV7 plays a vital role in directing DSB repair pathway choice. This finding, as well as REV7’s involvement in chemosensitivity, triggered a renaissance of sorts for REV7, stimulating investigations into its potential therapeutic value. This value has been further highlighted by a wealth of recent studies showing that REV7 dysregulation, and particularly its overexpression, promotes carcinogenesis and mutagenesis. Moreover, REV7 loss sensitizes various cancers to DNA damage-inducing therapy, and REV7 can act as a biomarker to inform therapeutic agent choice.

Despite these promising preclinical data, translating REV7-targeted therapies into clinical applications is likely to pose significant challenges. The development of additional effective inhibitors, along with the establishment of optimal dosing protocols and appropriate patient selection criteria, will be crucial first steps in addressing these challenges. As highlighted in this review, REV7’s involvement in multiple pathways related to genome stability makes it a complex therapeutic target, necessitating thorough evaluation of potential off-target effects in preclinical models. Furthermore, since REV7 expression and function vary across different cancer types, research is needed to elucidate the mechanisms by which tissue-specific contexts influence REV7’s role in cancer progression and therapeutic resistance.

**Figure 10:**
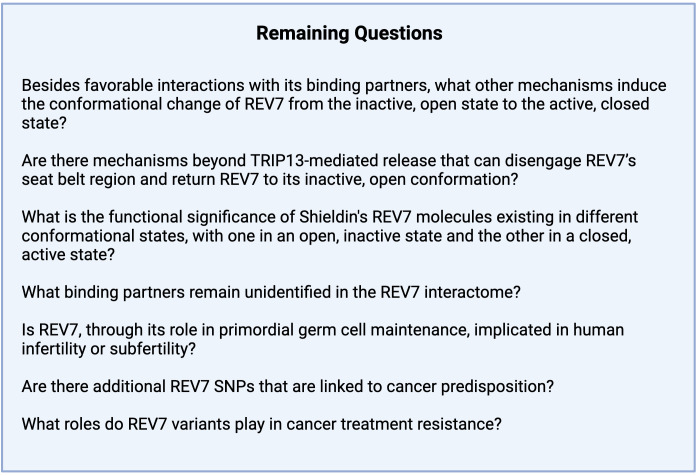
Remaining questions.

Many questions about the integration of REV7 and its binding partners into nuclear processes remain (**Figure 10**). Future studies will undoubtedly identify uncharacterized interactors and uncover additional features of this enigmatic protein. These investigations should help clarify how REV7 functions in its seemingly opposing roles as both an agent of mutagenesis and a steward of genome stability.
